# Stroke survivors’ telemedicine experiences during the COVID-19 pandemic: A phenomenological investigation

**DOI:** 10.1177/20552076241281464

**Published:** 2024-10-07

**Authors:** Barbara Kimmel, Annette Walder, Anette Ovalle, Chethan P Venkatasubba Rao, Sean I Savitz, Mark Goldberg, Steven Warach, Salvador Cruz-Flores, DaiWai M Olson, Jane A Anderson

**Affiliations:** 1 Houston VA HSR&D Center for Innovations in Quality, Effectiveness and Safety, Michael E. DeBakey Veterans Affairs Medical Center, Houston, TX, USA; 2 Department of Neurology, 3989 Baylor College of Medicine, Houston, TX, USA; 3 Department of Medicine, Baylor College of Medicine, Houston, TX, USA; 4 Institute for Stroke and Cerebrovascular Disease, UTHealth-Houston, TX, USA; 5 Department of Neurology and Medicine, UT Health Science Center at San Antonio, San Antonio, TX, USA; 6 Department of Neurology, Dell Medical School, The University of Texas at Austin, Austin, TX, USA; 7 Department of Neurology, 37316 Texas Tech University Health Sciences Center El Paso, El Paso, TX, USA; 8 Neuroscience Nursing Research Center, The University of Texas Southwestern Medical Center, Dallas, TX, USA

**Keywords:** Stroke survivors, secondary stroke prevention, self-management, telemedicine, underserved

## Abstract

**Purpose:**

This study aimed to describe stroke survivors’ experiences receiving telemedicine visits at the Lone Star Stroke Consortium during the COVID-19 pandemic.

**Materials and Methods:**

A qualitative descriptive phenomenological design was applied to gather patients’ telemedicine experiences through in-depth interviews, using a study guide. Audio-recorded interviews were conducted via ZOOM and transcribed verbatim. Two independent reviewers used the Giorgi descriptive method to analyze the data and search for the essence of stroke survivors’ follow-up telemedicine experiences during the COVID-19 pandemic.

**Results:**

Fifteen underserved patients were recruited: mean age, 51.8 (15.7), and 9 (60%) females. Three themes emerged: (1) vivid memory of the stroke acute phase, (2) poststroke care experiences, and (3) perceived telemedicine experiences.

**Conclusions:**

The phenomenon of follow-up telemedicine visits during COVID-19 pandemic, as experienced by the stroke survivors, was positive. It showed patients’ improved care access for poststroke visits. Telemedicine was satisfactory, except where the full medical exam was needed. Study findings highlight the individual approach was important, as well as the need for reliable internet and training to improve patients’ technological skills. A hybrid approach for post-pandemic follow-up visits (in-person and telemedicine) was recommended by stroke survivors. These findings suggest that telemedicine is feasible and effective for poststroke care. Additional strategies are needed to improve future telemedicine integration into the continuum of care.

## Introduction

The COVID-19 pandemic was catastrophic, causing loss of life and anxiety and depression globally.^1^ Social isolation and uncertainty about the pandemic affected how the world's population approached regular health care needs. Most health care provided outside the hospital was limited to urgent need. Routine care was postponed or terminated or, if available, provided using telemedicine.^2–4^ The pandemic was especially challenging for individuals with cardiovascular diseases.^5,6^ The pandemic provided a “stress-test” for the world's healthcare systems, with not all of them passing it; and, therefore, studying the response seems to be a worthwhile “first-line” exercise, with possible lessons for the future.

In 2019, the American Heart Association/American Stroke Association (AHA/ASA) released an updated policy statement to guide effective implementation of the full continuum of stroke systems of care.^7^ However, shortly after release, the COVID-19 pandemic emerged, creating enormous challenges and strain on health care services worldwide, disrupting established stroke systems of care.^8^ Unfortunately, necessary public health measures to reduce COVID-19 infections resulted in lockdowns and social distancing that prevented access and delivery of necessary health care services. In relation to stroke, patients’ access to lifesaving therapies was limited, as well as access to guideline-concordant follow-up care after stroke, including rehabilitation, effective stroke risk factor management, and reintegration with social and economic resources.^9^

To help mitigate limited access to stroke care during the pandemic, the AHA/ASA released temporary emergency guidance to US stroke centers in April of 2020.^10^ The major recommendations were to follow treatment guidelines to ensure appropriate stroke care was provided during the crisis. This was especially important, as previously reported needs of stroke survivors and their caregivers are complex while moving from acute care to rehabilitation and transition to home care. Strategies included increased use of tele-stroke networks for acute stroke evaluation and treatment and implementation of telemedicine solutions for continued delivery of stroke follow-up, secondary prevention, and rehabilitation.^11,12^

To assure continued delivery of stroke follow-up care, including rehabilitation and secondary stroke prevention, the medical community was advised that telemedicine should be employed, where possible, to effectively carry out care and implement infection control measures. In addition, to support implementation of telemedicine for patients during the pandemic, reimbursements were established by the US Center of Medicare and Medicaid.^13,14^ The uptake in telemedicine use emphasized its benefits, especially for the underserved populations, by increasing access and better medical management.^11–16^ However, evidence is insufficient that telemedicine assures poststroke care continuity, especially within the first six months following stroke and under less structured clinical conditions.

We believe that the knowledge gap exists: No qualitative studies were published to directly report the impact of telemedicine during the COVID-19 pandemic on stroke survivors’ experience with poststroke care. To address the knowledge gap, stroke patients’ experiences should be closely explored, particularly for stroke survivors living in rural and low-income communities. These experiences to some extent were previously explored in the literature, but not using the phenomenological approach and not during COVID-19 pandemic. For this reason, our focus was to give patients the opportunity to express their feeling about experiencing stroke during the pandemic and their experiences of being treated during the acute and follow-up phases during the pandemic and lockdowns. Sharing these experiences allowed patients to recall events in more detail and in the context of their life-changing event, including their disability. In addition, pandemic-related stress and anxiety could have a negative effect on stroke survivors’ emotions. Therefore, this approach offered a unique viewpoint and added depth to the study from the patients’ perspective surviving stroke during the pandemic and their telemedicine experience while the pandemic was still present. Understanding patients’ experiences will help healthcare providers to design and improve interventions via poststroke telemedicine care.

Although the COVID pandemic does not have an equally negative impact on healthcare now compared to what it had during the peak of the pandemic, lessons learned from this study regarding poststroke follow-up experiences using telemedicine will still remain valuable. Among other delivery strategies, telemedicine plays a crucial role not only in optimizing stroke acute care and preventing complications but also in possibly improving follow-up poststroke care and patients’ outcomes, especially via telerehabilitation. These aspects should be further evaluated in large-scale randomized control trials. The fact that 2024 COVID infections seem radically different from the 2020 pandemic does not preclude emergence of a more dangerous variant in the near future.

The aim of the study was to explore stroke survivors’ experiences of telemedicine visits at the Lone Star Stroke Network during the COVID-19 pandemic.

## Methods

A qualitative study design was applied, using descriptive phenomenology to capture the core lived experience of stroke survivors and their caregivers (if applicable).

The phenomenological approach was applied because it allowed participants to describe, in their own words, their experiences of receiving follow-up care by telemedicine.^17–20^ Creswell^20^ summarized the phenomenological approach as one in which individual is making sense of personal experience and is acting without assumptions. In other words, this approach is without any judgment; and it allows the researcher to identify shared experience^21^ and consequently obtain the essence of the experience, which, ultimately, is the study goal.^22^ We used the transcendental approach with a bracketing method (van Manen, 1990: Husserl, 1962),^17,23^ setting aside any prejudgments regarding our investigation.^18^

### Eligibility criteria

We screened potential participants 18 years or older, who experienced stroke during the COVID-19 pandemic, and who were followed-up by telemedicine for their poststroke care.

### Recruitment

The study was implemented within the Lone Star Stroke Consortium (LSSC), a research network of academic medical centers (hub sites) linked to affiliate community hospitals and clinics (spoke sites) across Texas.^24^ Institutional Review Board approval was obtained from the LSSC hub sites.

To achieve common understanding of patients’ experiences, we recruited a purposive sample of participants who met inclusion criteria, from five LSSC affiliate spoke sites that serve higher numbers of stroke survivors from rural and low income-urban communities.^25^ Participants were not recruited on a representative basis, but rather based on their demographic characteristic and knowledge of the “under investigation phenomena.” These participants resided in geographically spread areas of Texas with limited access to care facilities to due to lack of private or public transportation and/or inability to receive poststroke care, including rehabilitation services. In addition, we selected participants who, due to the pandemic, decided to stay away from in-person treatment facilities and used telehealth services instead.

This approach does not require data saturation as *saturation* is not part of the phenomenological method but a term used and applied in grounded theory.^22,26–31^

Participants were referred for study by their treating provider. In addition, discharge lists of participants diagnosed with stroke or transient ischemic attack (TIA) who also received follow-up telehealth services were reviewed for possible participation. The diagnosis of ischemic stroke or TIA was determined based on the stroke and TIA IDC-10 codes.

A research assistant (RA) established initial phone contact with the eligible patients to introduce the study, explained consenting procedures, and informed patients about the in-depth interviews and financial incentives for participation. Patients indicating interest in the study were contacted by phone to complete an electronic consent form. Over nine months, 20 eligible patients signed the consent form. After multiple attempts, we were unable to reach four participants to schedule interviews; and one patient declined participation. As a result, 15 patients participated in 60-min in-depth individual interviews.

The interview sessions were conducted virtually using ZOOM technology. English-speaking patients were enrolled and instructed on the in-depth interview process and on how to use Zoom^©^. We adjusted to patients’ availability and scheduled interviews to make things as convenient as possible. The first author, who is employed as an instructor at the research study institution, has a doctoral degree in health prevention and behavior sciences and specializes in qualitative studies, conducted the individual interviews. Using the individual patient interview guide, we addressed attitudes, perceptions, barriers, and facilitators to telehealth services for postacute stroke care, as well as education and self-management to prevent another stroke and how to best control stroke risk factors. All interviews were digitally recorded and transcribed by the Lighthouse of Houston transcription services. Upon completion of the transcriptions, the files were placed into a password-protected study drive for analysis. Transcripts were not returned to participants for comment or correction, but participants were informed that they can receive transcripts upon request.

### Data collection and interview guide

Demographic information was collected for each participant before initiating an interview session and is reported in Table 1. The principal investigator (PI) led all sessions with the RA, who collected field notes. Each interview was initiated with a short introduction between the PI, RA, patient, and patient's caregiver (if applicable). The pandemic restrictions were still fully in effect, and all patients connected via Zoom from their homes. Privacy issues were discussed, and concerns raised by the patient were addressed. Participants were informed about LSSC Research Network mission, goals, and reasons for conducting this specific research. An interview guide was developed previously, pilot tested, tailored, and adopted to this study to specifically capture telemedicine follow-up lived experience during the COVID 19 pandemic.^32^ For the complete Interview Guide, please refer to Appendix 1.

**Table 1.** Participant characteristics.

Characteristics	Freq (%)
Age years mean (std)	51.8 (15.7)
Gender	
Male	6 (40)
Female	9 (60)
Race	
White	12 (80)
Black/African American	3 (20)
Ethnicity	
Hispanic/Latino	5 (33.3)
Non-Hispanic/Latino	10 (66.7)
Date of Stroke Event	
2020	11 (73.3)
2021	4 (26.7)
Stroke Type	
Ischemic	11 (73.3)
Hemorrhagic	4 (26.7)
Rehabilitation	
Inpatient	4 (26.7)
Outpatient	4 (26.7)
None	7 (46.6)
Employment	
Not Employed	6 (40)
Employed/Self Employed	3 (20)
Disabled	0 (0)
Retired	6 (40)
Insurance coverage	
Yes	14 (93.3)
No	0 (0)
Missing	1 (6.67)
Caregiver	
Yes	3 (20)
No	12 (80)

The initial question was structured to first capture patients’ lived experiences of a stroke event during pandemic: “Please tell me how it was for you when you started to experience stroke symptoms*.*” The purpose of the opening questions was to make patients comfortable and allow them not only to recall their acute event but also to share the experiences of suffering a stroke during the pandemic and express questions or concerns they might have prior to discussing their specific telemedicine experiences.

Generally, questions were structured to capture patients’ descriptions of their lived experiences during hospital stay and after being discharged home. The interview guide included questions to capture patients’ experiences with postacute stroke follow-up care using telemedicine and perceptions of the concept “stroke risk factor management.” Staying truthful to the phenomenological approach, we made sure to apply bracketing concept by removing our personal experience while conducting interviews^20^ and when performing analysis. As defined by Husserl,^19^ bracketing makes it possible to “unpack” the phenomena under the investigation, which results in only the true factual experience to remain. We also recognized our own worldview and the way we adopted in the research process.

When patients’ responses to any question were ambiguous, follow-up probing questions were used to elicit a more in-depth response. For example, “Can you tell me more about….?” In addition, prompts and probes were used to provide comfort and encouragement when appropriate. Our overall goal during the interview session was to ensure participants that their voices were fully represented and that they were comfortable sharing their life experience.^33^ Each interview lasted for approximately 1 h, and participants received $50 compensation in the form of a gift card.

### Data analysis

Participants’ sociodemographic characteristics were analyzed descriptively and are presented in Table 1. Interviews were digitally recorded and transcribed verbatim. The Giorgi method was applied to describe how the individual “made sense of a given phenomenon.”^34^ This method aligns closely with a descriptive phenomenological approach, and it was developed based on a phenomenological philosophy. In contrast to a purely empirical approach, Giorgi's broader phenomenological method leads to more suitable psychological development, allowing individuals to make sense of personal experience and act without assumptions.

This discovery-oriented method is characterized by four major principles: it is descriptive, it uses reductions (meaningful units), it searches for essence, and it focuses on intentionality. As a result, a researcher can describe the essence of “lived experiences” based on the perceptions of individuals who lived through an event that occurred within a specific context. Participants’ voices are kept through the analysis without abstracting their opinion.

Two trained investigators independently read and re-read transcripts to obtain an overall sense of the participants’ description of experience, identified and reduced descriptions to the meaningful units and coded each transcript. Bracketing was applied and included discussion of biases and reaction to the data and how the biases might contribute to the interpretation of the interview content. Once initial coding was completed, the investigators engaged in an iterative consensus-building process to verify all similar and unifying codes they identified in their initial independent coding process. Detailed description of the coding tree is available upon request. Next, concepts were formulated from verified codes and categorized as emerging themes and subthemes. As additional concepts emerged with each analysis, new concepts were added using this constant comparison process until emerging themes were achieved.

When necessary, authors returned to the raw data. The themes were derived from the data, based on which phenomena were described.

Giorgi's descriptive analysis and the field notes allowed identifying what and how participants experienced as the “phenomenon under investigation” when answering the question: “What is it like to experience stroke and receive telemedicine follow-up care” during the COVID-19 pandemic?” Participants did not provide feedback on the findings.

## Results

### Clinical characteristics of the participants

Most patients experienced ischemic stroke and, despite having some insurance, almost half did not receive any poststroke rehabilitation treatment. Most did not have a caregiver, which complicated recovery. In terms of functional disability, all patients were able to walk; however, all but three suffered from several chronic illnesses such as hypertension, diabetes, and elevated cholesterol levels. Regarding modifiable behavioral stroke risk factors, 30% of the patient sample was obese; and several suffered from depression, anxiety, and stress. Over 50% reported lack of physical activity and problems with medication compliance.

Most patients had someone call 911 and arrived at the hospital by ambulance. Two had someone take them to the emergency room, and one did not go to emergency, even though they knew there was a problem: they had their friend take them the following day. Two who arrived showing mild-to-no symptoms were sent home. The average time patients spent in the hospital was 10 days, with a minimum stay of two to three days and a maximum of 30 days. Seven patients were discharged to home, with the remaining discharged to receive rehabilitation services. Cumulatively, patients participated in 42 telemedicine visits. Telemedicine visits were conducted mostly with neurologists, primary care physicians, and rehabilitation services. In addition, two patients were seen by a psychiatrist and two by a cardiology specialist. One also consulted with a rheumatology specialist.

### Themes

Three major themes emerged from the phenomenological analysis: (1) Vivid memory of the stroke acute phase, (2) Poststroke care experiences, and (3) Perceived telemedicine experiences.

#### Theme 1: “Vivid memory of the stroke acute phase”

Vivid memories of stroke survivors are common experiences. Patients described how the acute stroke profoundly changed their lives, physically, emotionally, and psychologically, particularly because the event occurred during the COVID pandemic. Despite the devastating event and COVID restrictions, patients still showed resilience while seeking care.

#### Subtheme 1a: Onset of stroke symptoms

Patients provided detailed accounts of the acute event that included patients’ fear, confusion, frustration, and uncertainly due to experiencing stroke during the pandemic. They also experienced confusion due to a sudden impairment of motor skills, such as talking, walking, using hands, or having tunnel vision. One patient was in a state of distress thinking that she was dying. For a 75-year-old woman (Patient 030), stroke was sudden and something that she had never experienced before and affecting her deeply on the emotional and social levels. She said:

First of all, I wasn’t even aware that something was happening. My husband had been gone to a business meeting. He walked into my office. He took one look at me and said, “Oh my God, we’ve got to call an ambulance.” … the ambulance arrived within 10 min…my husband told me what he saw was that one side of my face was dragged down or whatever, and when he walked in, I tried to speak, and I could not speak.

A 56-year-old woman (Patient 076) reported frustration due to an unexpected change in her condition, when she started to have numbness in her left side, noticed facial dropping and then dropped her phone:

. . .and I told my employer, my boss, that I was having a stroke. I knew the signs

and symptoms of a stroke… So, I hung up, and I called my husband, and I called 911 and told them that I was having a stroke. . ..

One patient (Patient 058, male, age 45) expressed his fear while experiencing stroke at work:

I knew that I was in trouble…. Like everyone's expression was very like scary.

#### Subtheme 1b: Experiencing the stroke during pandemic

Since the pre-pandemic period, patients experienced multiple challenges surviving stroke and poststroke care. Recognizing preexisting issues and also having a stroke during the COVID-19 pandemic created a different set of challenges and frustration. Some of them experienced stroke symptoms when awareness of the pandemic was just beginning to emerge, while others experienced stroke symptoms when the pandemic was widespread. Participants expressed hesitation in seeking help, especially in terms of deciding whether to call 911, due to fear of infection. Some indicated that a family member called 911 and they were taken to the hospital by ambulance immediately, despite their COVID concerns. One said,

Thankfully, I have a wife who loves me that said we’re dialing 911, and she dialed 911. I would have probably been hesitant to dial 911, and that needs to be a part of the training.

Another patient, a nurse, said that she remembered well lying on the floor on that day, thinking:

Having to go and going into the hospital during the COVID pandemic… and there was a panic inside of me. You know. Lord, God, I'm having a stroke, and then it's during COVID times. So, of course, we’re going to the nearest hospital.

New COVID protocols implemented by care facilities did not allow visitors, adding additional confusion and frustration, especially at the beginning of the pandemic. Patient 054, Female, age 49 said:

The pandemic, they were wearing masks and only certain people…. You know. We couldn’t have visitors, and everybody was worried, and so it was… It was difficult.

Patient 031 (male, age 71) also experienced uncertainty due to the same restrictions: “In April of 2020, at the height of COVID, they didn't allow anybody in the hospital. So there was no me going to the hospital.”

Later some hospitals allowed one person, and patients still felt like they were getting good care. For example, a 48-year-old woman noted

The ambulance came…. The next thing that I remember is hearing the ambulance back up in the drive, and then…. Then, after that, I was in the hospital for two weeks. My husband stayed there…but he was the only visitor allowed. So, no one else could visit. They only allowed one visitor.

#### Theme 2: “Poststroke care experiences”

In the second theme, we learned about patients’ poststroke care experiences after being discharged home. The discharge process from hospitals should prepare patients and caregivers to adhere to complex discharge instructions and follow-up with postdischarge services. However, visit coordination, risk factors control, and access to rehabilitation, as expressed by our study patients, were challenging, particularly due to COVID pandemic restrictions.

#### Subtheme 2a: Care coordination challenges

Most patients discharged home had a checklist with instructions. However, it was challenging for them to adhere to all recommendations and coordinate visits. Several patients expressed frustration about difficulties with making appointments and anticipating care delays due to the pandemic. Patients expressed concerns and inability to follow all discharge instructions and coordinate multiple follow-up visits with doctors. For example, (Patient 018, female, age 33) said: “I think that the most overwhelming part of this whole process to me was the number of follow-up doctors to sort through.” She also said: “This doctor wants to see you here, and my brain was in such a fog that I just felt like that I don’t know who I'm talking to.”

Notably, Patient 056 (female, age 35) described her challenges with insurance and seeing a doctor for poststroke visits:

Because of my insurance, not everything…because I wanted to see the doctor like really…like I wanted to see the doctor like oh, I’m going to see the doctor soon, but it took three months (PHI). So the papers were not … what was it? The referrals for the… for the…yeah, for the specialists or for the therapy were not there.

She also later commented:

So I had … so I knew that I had to you know. I had to start… so me by myself because my papers were not in, the referral was not in, I had to start little by little doing things so that I could better myself.

#### Subtheme 2b: Rehabilitation challenges

During the interviews, patients expressed their feelings about poststroke care continuity involving personal rehabilitation plans essential for successful recovery. Rehabilitation services (inpatient and outpatient) were offered to patients despite the pandemic. When asked about any shortage of staff in rehabilitation hospitals, both Patients 067 and 070 noticed shortages but did not feel they affected their therapy significantly. Patient 067, a 46-year-old man, remembered days when his therapy was cut due to lack of personnel:

So there were days like, especially on the weekends, that there was just no therapy at all because there weren't enough folks there. I'm in no way complaining. I feel in this situation that I was fortunate and blessed, and I'm grateful for the things that I did receive. Because I know that this condition…. In the hospital, I saw that, one, this condition can go a lot of ways.

Patients also commented on receiving face-to-face therapy (despite COVID restrictions) before having an opportunity to follow-up with telemedicine visits. Patient 070, a 48-year-old woman commented,

I got therapy right away and was in therapy every day. Well, not every day but 5 days a week,

and patient 031, a 71-year-old man, remembered his care being good:

One thing that made a big difference was the fact that at any time that I needed something or someone, they were there. If I pushed that button for the nurse, those…. That first 5 days, they were…. I mean they were in that room within 30 s.

In contrast, a very powerful statement related to inpatient rehab was received from a 56-year-old woman who provided a thoughtful account of her experience in a dysfunctional health care facility that had obvious staffing issues due to pandemic restrictions, a lack of professionalism and poor bedside manners. She stated:

It was terrible for me to just watch it, and being a nurse, it was just…. It was like a nightmare. It was truly a nightmare. The staff had no…. Really…. The majority of the staff had no concern as far as the patient needs are concerned…. You were basically sometimes just left there. You know. To fend for yourself, and it was terrible. It was terrible to where I had to contact the administrator of the hospital. At one point when I was there, I even had to dial the hospital operator to get assistance in my room.

#### Theme 3: “Perceived telemedicine experiences”

The COVID-19 pandemic radically modified care delivery for stroke. Recommendations were made for remote follow-up, including rehabilitation and self-management strategies for risk factor control. When engaging in a telemedicine encounter instead of face-to-face poststroke visits, patients shared their experiences describing what really worked for them and why telemedicine makes it possible for continuity of care, including follow-up visits with specialty providers at a convenient time in the comfort of their own home. Conversely, barriers to telemedicine visits were identified regarding lack of person-to-person touch and technological challenges.

#### Subtheme 3a: Advantages with telemedicine encounter

Majority of patients liked improved access to health services they received giving them a convenient option with less aggravation than in-person visits and eliminating geographic barriers. Specifically, telemedicine visits eliminated the need for travel and parking costs. Patient 069, a 68-year-old man, expressed his view:

It may be that, sitting in my kitchen, I was more able to focus on the problem at hand rather than worrying about the manipulation of myself to the hospital and having all of the pieces and having my notebook of notes, and I was more relaxed.

Patient 067 also appreciated telemedicine visits because he was able to have multiple devices and materials easily accessible, “. . . whereas, coming into the office, I’ve got to have…. I’ve got to think about how that I'm going to bring the things that I need to. . . .”

Many patients felt safe with the telemedicine option for their follow-up visits, especially during the pandemic. Patient 061 said:

It was like she was there, but she wasn't there. You know. I could talk to her and without catching anything. Not that she had anything, but you know. It was good. It was good that it was offered to me. So, and it was safe.

Moreover, due to multiple lockdowns to prevent spread of COVID-19, participants noted that having telemedicine visits bridged gaps in “care continuity” and helped them to improve care coordination among providers such as primary care, cardiology, mental health specialties and physical therapy. Patient 071 recounted being able to have both cognitive and physical therapy via telemedicine:

During those times, you really couldn’t get out to go and have the physical therapy, so I had to do most of that at home, and the cognitive therapy was mostly done on my own here also. Just little basic exercises: trying to remember things, using flash cards, using…. Just testing myself daily trying to remember what I did the day before and trying to organize things.

Patient 061 said, “I’ve had tele-care with the psychiatrist, with my neurologist, and with my ENT primary care doctor.”

Participants also noted that telemedicine visits allowed providers to conduct quick clinical assessment of their physical disabilities as well as an evaluation of the home environment. Patients received advice on what changes they should make to make their home stay more conducive to the specific situation poststroke.

Patient 076, who, after her stroke was home with a spouse at work, thought the telehealth was “a wonderful thing” and was grateful that her primary care provider had set up telehealth visits at a time convenient for her and ensured that she was taking all her medications. She appreciated that, during, her telehealth visits:

They can actually see me. You know. How I was feeling. How I was doing. How the medication was working, and even with my stroke, with my specialist at __, you know. How was my arm. You know. Were there any improvements with my arm movement, with my legs. The stroke affected my left side. How was my blood pressure doing. To make sure that my medication was effective and that I had refills.

In terms of time commitment, patients were quite satisfied and reported that their needs were addressed with an ample amount of time being spent on the visit. The video links were sent to patients in advance, and any technological issues were discussed before scheduled visits. Appointments were on time, or patients were called if there was a delay. As Patient 069, a 68-year-old man said,

The time that the doctor spent with me, I felt that okay, and this is a personal perception, the doctor was more at ease and was willing to listen and share and speak to me about my issues.

Patients felt telemedicine visits were convenient. Both patients and caregivers did not have to miss work. Patient 067 said: “Like, I could have it early in the morning. She didn't have to be here missing work and stuff. So she could, yeah. So there is that, you know, and not just driving over there.” They can have their advocate with them during the visit.

Furthermore, patients reported that telemedicine visits helped with a direct caregiver's or family member's communication with providers. Patient 067 thought that “it worked out great,” and that it was great to be able to have someone else listening and ask questions they wouldn’t have thought to ask:

Because here is what was awesome was that I asked people to come to visit me at the house, obviously, and so I was also able to have my ex-mother-in-law, who is an RN . . . . So she was kind of familiar with some things. So she was able to sit in on the conversations with me, even though my mother wasn't there. So she was able to listen and, like, give me some advice about how to proceed with those types of things that I needed and talk about some medical terms that I might not have been able to understand.

Patients’ comments on self-management and risk factors control constitute a good contribution to address unique vulnerabilities of individuals with chronic conditions and strategies to overcome these during care transition and recovery. Furthermore, among multiple benefits of telehealth, patients’ empowerment is an important component in promoting effective patients’ recovery. Self-management in this context encourages patients to self-monitor their treatment plan, enhances communication with the providers, and sets realistic goals contributing to improved outcomes*.*

Patients shared their experience regarding stroke risk factor management in the self-management framework. The lack of face-to-face visits to learn more about self-care and what it means to successfully manage stroke risk factors and make necessary lifestyle changes was particularly challenging. Patients liked the opportunity to discuss changes to their diets and review exercise routines and better medication compliance strategies. Patients shared their experiences about goal setting for very specific risk factor control. Providers encouraged them to exercise daily, prepare healthy meals, drink more water, reduce stress, and maintain their medicine schedule. Patient 061, a 66-year-old woman said: “I incorporated more vegetables, turkey. You know. I don’t eat a bunch of red meat. Sweets and stuff. I went off the soda. You know, but I lost weight.”

#### Subtheme 3b: Disadvantages

While there are many benefits of telemedicine encounters identified by our patients, some aspects of the virtual visits were perceived as “barriers.” This was prominent when patients shared their experiences about their social and emotional connection during visits, as well as technological barriers. Physical exams were limited, and patients preferred the in-person visit physical exams to be performed immediately after being discharged home. A few patients also found in-person visits more mentally stabilizing. One woman, a 33-year-old woman, stated:

I had it on my cell phone, and so like trying to prop my cell phone up and do my fingers and touch my toes and everything was difficult, and I felt like that that call should have been done in person.

In addition, patients shared concerns that sometimes the home environment created distractions, preventing them from addressing all their problems with the doctor. They also shared concerns that technology distracted communication due to unstable internet connection or other problems. In terms of limited assessment capabilities (i.e., vital signs and in-depth neurological exam), patient 054 (a 49-year-old woman,) said:

Well, it was easier to see me and how I was doing versus going in person where they could see how I was walking and my balance and stuff. It was better in person versus being on telemedicine.

The most serious technological challenges were due to Wi-Fi service, connection, and availability, as well as platform issues. Patients would lose the connection, and then the doctor would call them on the phone to complete the visit. Various platforms such as Zoom, My Chart, and FaceTime were used, and some worked better than others. For example, Patient 063 said: “The only thing was, like, my neurologist, like on his line, I couldn’t hear him. So he just, like, transferred over to a phone call instead.”

In summary, the goal of this study was to gain understanding of stroke survivors’ lived experiences during the Covid-19 pandemic related to their telemedicine follow-up care. Our initial enquiry was related to patients recalling the acute phase of their stroke during the pandemic. Patients felt fearful, confused, frustrated, and uncertain due to experiencing stroke during the pandemic. In essence, they perceived stroke as a debilitating disease and the COVID-19 pandemic as an additional challenge while receiving poststroke care. The perception of follow-up telemedicine visits during the COVID-19 pandemic, and as experienced by the stroke survivors, was positive. It showed improved care access and several advantages of the telemedicine encounter. Patients felt that telemedicine was satisfactory, unless a full medical exam was needed. The essence of the experience also included patients’ perception of various technological barriers and absence of face-to-face patient–doctor interactions.

#### Patients’ follow-up care recommendations post-pandemic

At the end of each interview, we asked patients regarding future recommendations and visit preference after the pandemic as an important contribution for future studies and lessons learned for postpandemic care models. Since we explored patients’ experiences during the pandemic, we felt that it would be valuable to report what patients shared regarding utilization of telemedicine in postpandemic care. We asked for these recommendations that were not directly the study aim but patients’ suggestions may contribute to the literature on poststroke, and the way care can be affected similarly to what happened during the COVID pandemic. We understand that these suggestions need further investigation to establish a direct link.

Patients recommended to be seen face-to-face immediately after a stroke and have telemedicine visits for their follow-up care, as suggested by Patient 018 (female, age 33): “We can do a telemedicine call, but if you do face-to-face, he can listen to your heart, and he’ll get a blood pressure check that day, and that could tell us more information.” Patient 061 also felt there should be a choice of the types of visits offered, depending on their disability and/or lack of transportation: “Well, I say that because I don’t drive, of course, but if my sister couldn’t take me, then I would…. You know. Then, I would have to go to the video.” Concerning the general stroke follow-up visits for risk-factor control issues, our participants felt that they should have a choice, as stated by Patient (015): “… if they ask me do you want to do the teleservice or do you want to go in person. I think that…. I think that we should decide.” Finally, participants expressed interest in a hybrid poststroke care visit model when describing doctor–patients’ interactions. Patient 069 (male, age 68) felt that telemedicine is great because you can sit at your table and do it, but that there is a psychological benefit to face-to-face visits:

You feel that you are cared for better if there is a human being there rather than a screen… well, you can't read my blood pressure. You can't feel my heart beating…So, comparing the two, I would say that face-to-face was far and away better, but the Zoom visits, the telemedicine visits, if no physical interaction was necessary, if we just wanted to talk, then they’re effective and pretty good.

## Discussion

Understanding stroke survivors’ lived experience during the COVID-19 pandemic can inform researchers and clinical providers how to improve the quality of neurological care and effectively reduce the overall burden of stroke. This is particularly important as telemedicine, conceived as practice to use technology to remotely deliver care during the pandemic, also may help to efficiently deliver much needed poststroke follow-up care.^35^

With respect to the acute phase care experiences, our finding agrees with literature reporting that factors related to recovery from acute stroke involve restructuring and adaptation of physical, social, and emotional aspects of individuals’ lives.^32,36^ Moreover, experiencing stroke during the COVID-19 pandemic created additional challenges seeking hospital care and after discharge home, as the fear of viral infection altered in many ways patients’ and caregivers’ decision-making.^37^ Our participants expressed frustration, fear, and a lack of clear understanding how health care facilities would operate during the pandemic. Although no studies addressed that in direct relation to stroke, we found several articles exploring how the pandemic disrupted health care systems that reported similar conclusions. For example, Moore et al.^38^ confirmed that, within primary care settings, patients experienced delays and avoided care due to fear of COVID-19 infection. Our results revealed that patients who decided to seek hospital care experienced difficulties because no visitors were allowed at facilities, resulting in a sense of isolation, anxiety, and loneliness. As also reported by Mukhti et al.,^39^ caregivers were equally affected, being faced with extra burden through different experience. Furthermore, postdischarge rehabilitation (physical, occupational, and speech), which is the most important element in the first three months of patients’ recovery, was also adversely affected due to personnel shortages and challenges to visit scheduling. We consider this as a significant finding, especially regarding future clinical recommendations to improve follow-up care. Delays and forgoing care were also reported by Gonzalez et al.^40^ as constituting a major cause of increased mortality rates during the pandemic. Additionally, a high number of deaths associated directly with the COVID-19 virus were observed in increased mortality rates due to other causes as a secondary result of the pandemic.^41^

Regarding the telemedicine encounter phenomenon, our findings revealed multiple advantages and disadvantages of using telemedicine for poststroke follow-up care. A majority of patients liked improved access to health services received in the comfort of their own home and at the same time reduction of travel times and transportation and parking costs. The rapid transition to telemedicine during the pandemic allowed patients to feel safe and avoid physical contact with other patients and health care providers, which emerged as 1 of the phenomena under our investigation.^3^ Telemedicine visits were convenient and efficient and provided continuity of care coordination, since stroke survivors’ follow-up care requires multiple consultations with various specialists and can be challenging. For example, as patients transition from hospital or inpatient rehabilitation to the community, they may not be fully prepared to continue their rehabilitation programs at home. The main issues can be lack of clear instruction postdischarge, lack of insurance, or insufficient care services. Therefore, more support for patients is needed, as well as additional research should be conducted to improve transition and provide stronger support to meet recovery goals*.* These findings are consistent with other studies’ results, as reported by Olszewski et al.,^42^ Knepley et al.,^43^ Pappot et al.,^44^ and Holtz.^45^ In addition to increase motor, cognitive, and speech function, patients made positive comments on self-management coaching provided by telerehabilitation during the pandemic. Patients reported successfully using the telemedicine platform to discuss behavioral risk factor control, such as setting up specific self-management goals including daily exercise routines, improved diet, reduced stress, and generally finding the best ways to improve quality of life during the pandemic and beyond. A similar finding was reported based on the systematic review conducted by Knepley et al.^43^ and Hwang, Park and Chang.^46^ Authors concluded that, even though no clear evidence exists that telerehabilitation is superior to in-person therapy, delivery of therapeutic interventions, including self-management stroke support, via telemedicine is beneficial to stroke recovery. Overall, telemedicine appeared to improve people's control over their recovery. This was especially apparent in terms of care access, decreased wait times for appointments, no need to commute to the doctor's office for those in remote areas and decreased cost due to telemedicine. Timely clinical evaluation and self-management of controllable behavioral stroke risk factors also contributed to their recovery.

Regarding disadvantages, our findings confirm, as was previously reported, that loss of the person-to person touch and impossibility of receiving a full neurological exam (including reliable vital signs measurements), especially immediately after discharge home, might be a barrier to telemedicine use. Additional barriers from our patients’ perspective were associated with technological challenges such as unstable internet connection, unreliable WI-FI availability, and various other platform issues.^44^ It is also worthwhile to mention that the telemedicine experience may not be the same for everyone. Health disparities such race, income, education, access to health services, and geographic location might adversely affect stroke recovery. Telerehabilitation can address some of these disparities, but only for people who have access to reliable devices and broadband internet. For that reason, more emphasis should be given to reduce these disparities by expanding healthcare services to these underserved populations.

### Strengths and limitations

To our knowledge, this is the first study following rigorous application of phenomenological methodology conducted with stroke patients during the COVID-19 pandemic, with the major objective to learn about their lived experience as they transitioned through the stroke continuum. In addition, the value of this specific qualitative approach lies in its ability to have patients’ perspectives about their encounters with digital health care systems and care teams, as well as to come up with recommendations for the future (post pandemic). We strove to enquire about patients’ experience during the stroke acute phase, captured experience with telerehabilitation and telemedicine home visits and also included self-management behavioral support aspects. This was a major undertaking, as patient screening, recruitment, and interviews were conducted during the lockdowns and even before the COVID-19 vaccine was available. We were successful in reaching our recruitment goal, and our sample size was demographically diverse across five research sites. We recognized that, while patients confronted enormous challenges having a stroke during the pandemic and faced hospitalization and postdischarge difficulties while dealing with their expectations for recovery during the pandemic, they were still willing and able to share their experience for our study. The findings of this study add to the body of knowledge about how to improve and restructure poststroke care across the stroke continuum, including a strong telemedicine infrastructure for our health care stroke facilities.

However, our study had several limitations. It focused on poststroke patients’ experience only and did not include the experience of caregivers. Experience of providers, including medical doctors and rehabilitation and behavioral therapists, is equally important and should be included in future studies. We are in the process of publishing results from another study centered on identifying barriers to and facilitators of telerehabilitation services among providers, stroke survivors, and caregivers located in the same LSSC Network. The results of this study will be published this year. In addition, we are aware that the study used only the ZOOM platform. We are unsure whether our results would be the same if other modalities had been used and with a population with a higher level of digital literacy. This study is also limited by including patients from only five academic institutions in a single state. Therefore, the results may not be generalized to the population at large. Although we learned patients’ perspectives on the rapid transformation at participating health care centers adopting telemedicine, we cannot generalize these findings to other medical facilities, such as those located in the more remote geographical regions with very limited internet connectivity. Finally, the study was conducted during the pandemic when multiple lockdowns took place for more than 12 months. There is a possibility that these specific circumstances may have altered patients’ perceptions while sharing their personal experience and, consequently, have affected study results.

### Future direction

On the basis of our study findings and previous studies, (prepandemic), stroke survivors perceive telemedicine as a useful system to receive poststroke care.^47^ Specifically, as previously stated, follow-up routine visits with specialty providers and well-designed telerehabilitation services with trained therapists qualify to be accomplished by this modality. Our patients engaged with telemedicine and recommended to adopt this model in postpandemic care. However, even though transformation of care occurred in study sites quite quickly, based on patients’ comments, clinical practice needs additional development to satisfactorily implement telemedicine to benefit patients. In addition, even though these matters did not come up as a major theme, we heard from patients that privacy and security issues and better communication between important stakeholders should be further improved.^48^ Finally, for faster adoption of telemedicine, systems transformation should include rapid adoption of health insurance reimbursements of telemedicine visits as a part of standard care.^49–52^

## Conclusion

Having a stroke during the pandemic created unexpected challenges that transformed poststroke care delivery. Patients’ lives dramatically changed, and because of the pandemic, telemedicine as a standard of care was rapidly implemented. Due to massive expansion of telemedicine application, people became more familiar with the technology and its adaptation and in turn increased the applicability of telemedicine. Stroke survivors expressed that telemedicine had not only many advantages but also created barriers, especially due to technology. Patients perceived that the individual approach was most important in dealing with challenges, as this is critical to receive good-quality care. Positive changes occurred while using telemedicine, including easier access and care continuity. However, in cases where a full medical exam was needed, patients’ preference was face-to-face visits. In term of barriers, more reliable internet, better communication platforms, and additional training of patients and providers are needed to ensure more efficient transformation of the system. Moving forward, patients recommended using a hybrid approach for postpandemic visits, including in-person and telemedicine visits. More studies are needed to assess long-term sustainability of telemedicine for stroke survivor's post-pandemic across the United States.

## Appendix 1. COVID-19 Stroke Survivors—Patient's Individual Interview Guide.

**Table:**

**Introduction**	Thank you for agreeing to participate in this interview. Your participation is completely voluntary. Today we will discuss your experience, and your responses will help us to better understand issues around postacute stroke care during the COVID-19 pandemic and poststroke risk factors management. We will be recording this session, and we will not use any identifying information.
**Purpose of the study**	The purpose of this interview is for you to describe your personal experiences surviving the stoke and your perspective on poststroke care to improve your health and to deal with your personal stroke risk factors using telemedicine.
**Warm-up question**	As a whole—please describe: How do you feel today?
**Next step**	Now we can start the session—we will ask set of questions; but if at any time you decide not to continue the interview, you may stop, as you are not under any obligations to complete the interview.
**Follow up open-ended questions**	**Part 1**. Tell me about how it was for you when you started to experience stroke symptoms. • Was this during the first wave of pandemic? • Did you call 911? • Were you admitted to the hospital right away? **Part 2**. Tell me about how it was for you when you first went home from the hospital (when applicable) after you had a stroke. • What were the challenges going back home? • What is was it like to be back at your own place? • Tell me more about your daily living after stroke. **Part 3**. Tell me about your poststroke care during the pandemic. What have you found to be the hardest part of your poststroke care? ○ Discuss if the doctors expressed the need to schedule follow up visits to prevent stroke hospital readmissions ○ Discuss social isolation aspects due to pandemic restrictions ○ Discuss specifics of the follow up visits’ challenges: appropriate use of preventive medications, rehabilitation services, monitoring for anticoagulants (when applicable), social support, or lack off. ○ Home health care during pandemic • What is the easiest part of poststroke care for you?
	When applicable ask the patient: Describe your experience of having follow-up visits with your doctor over the video camera while you are at home: • What was the best part of seeing your doctor on a video camera while at home? • What the easiest part of seeing your doctor on a video camera while at home? • What was the worst part of seeing your doctor on a video camera while at home? • What was the most difficult part of seeing your doctor on a video camera while at home?
	Other aspects: • Time spent with the doctor • Technology challenges • Distance traveled • Physical restrictions • Presents or absence of caregiver (when applicable) • Privacy while on the video camera with your doctor • Was there any part of your experience while on the video camera with your doctor where you felt you could not express your concerns or ask questions? • Was there any part of you follow up visit with your doctor, while on the video camera, that was not provided that your doctor normally provides when you see them in-person?
	**Part 4** Tell me what managing stroke risk factors means to you? – How would you describe or characterize stroke management? – Were you taught in stroke education, how to set health behaviour related goals and their achievement?
	Tell me more about your healthy lifestyle habits after stroke: • keeping physical activity, healthy diet, quitting smoking and restricting alcohol consumption, adequately hydrating avoiding excessive negative messaging, getting adequate sleep, maintain social networks, and attending virtually preventive online classes.
	Please provide an example of the plan in one particular area that you decided to work on and how you were able to get it done. Please let me know if there is anything else that you would like to add too this interview that was not covered yet during our discussion. THANK YOU FOR YOUR PARTICIPATION!
